# Sweating and Anæsthesia

**Published:** 1911-03-04

**Authors:** 


### ANÆSTHETICS

### SWEATING AND ANÆSTHESIA.

Sweating occurs under two conditions during anæsthesia. In the first case it is very often seen during the induction stage, when the patient is breathing stertorously, and it is then usually associated with a flushed warm skin. In the other case it occurs late in the anæsthesia, and is entirely different in type from the former. The skin is then generally pale and very cold, and the sweat appears in beads all over the body, more particularly on the forehead.

### The Cause of Sweating.

Physiologists tell us that the blood pressure has nothing to do with the secretion of sweat, but whether this be so or not these two forms of sweating occur, the one when the blood pressure is high, and the other when it is low. The first form is particularly liable to occur in fat, heavily-built people, and is seen more particularly when ether is the anæsthetic used. It is more likely also to come on when there is some degree of cyanosis. This form is associated with marked muscular action, and this is the reason why some surgeons do not like ether as an anæsthetic, because they say that the preliminary stimulation re-acts on the patient later.

When a preliminary dose of one of the opium compounds now so frequently used as concomitants to general anæsthesia has been given, sweating is much more common. For example, when morphine has been used without atropine, sweating of the uncovered parts more especially is marked, with atropine in combination it is not so striking. With the newer opium derivatives, such as pleistopon and and pantopon, sweating may on occasion prove troublesome, the patient's shirt becoming almost saturated. This is especially the case when no oxygen is administered at the same time as the anæsthetic, and when open ether is given in combination with pantopon or scopo-morphine.

### The Importance of the Second Type.

The other form of sweating is of much greater surgical importance. It occurs when the blood pressure is low, and is a sign that shock or collapse will shortly supervene, and therefore it is a thing which the anæsthetist will always be anxious about, particularly if it occur early in a long operation. Its importance lies in that it is an indication of serious vascular disturbance rather than in the mere fact of its occurrence. It should always be a sign for which the administrator should be on the look out; as soon as it is observed steps should be taken to combat shock by injection of fluid into the system, and by raising the blood pressure with such drugs as pituitary extract. It is better, however, rather to prevent its occurrence by putting the fluid into the patient before the operation than by trying to treat the shock after the sweating has already occurred. For that reason the anæsthetist should examine his patient carefully before giving the anæsthetic, not merely with regard to lung and heart conditions but also with regard to blood pressure and liability to shock. This last is, of course, exceedingly difficult to estimate, but practice and experience will enable the anæsthetist to recognise, just as easily as the experienced surgeon does, those cases in which prophylactic measures should be taken to obviate any danger from shock.

